# Frequent detection of worm movements in onchocercal nodules by ultrasonography

**DOI:** 10.1186/1475-2883-4-1

**Published:** 2005-03-23

**Authors:** Sabine Mand, Yeboah Marfo-Debrekyei, Alex Debrah, Marcelle Buettner, Linda Batsa, Kenneth Pfarr, Ohene Adjei, Achim Hoerauf

**Affiliations:** 1 Department of Parasitology, Institute of Medical Parasitology, Faculty of Medicine, Bonn University, Bonn, Germany; 2 Department of Parasitology, Kumasi Center for Collaborative Research (KCCR), Kumasi, Ghana; 3 Department of Helminthology, Bernhard-Nocht-Institute for Tropical Medicine^3^, Hamburg, Germany; 4 Department of Microbiology, University of Science and Technology (UST), Kumasi, Ghana

## Abstract

**Background:**

Ultrasonography (USG) is known to be a suitable tool for diagnosis in lymphatic filariasis as the adult filarial nematode *Wuchereria bancrofti *in scrotal lymphatic vessels of infected men can be detected by the characteristic pattern of movement, the Filaria Dance Sign. In onchocerciasis, moving adult worms have not yet been demonstrated by USG. In addition the verification of drug effects on living adult *Onchocerca volvulus *filariae in trials is hampered by the lack of tools for longitudinal observation of alterations induced by potentially macrofilaricidal drugs *in vivo*. The present study was carried out to determine the frequency of detection of moving adult filariae of *O. volvulus *by USG.

**Methods:**

In an endemic region for onchocerciasis in Ghana, 61 patients infected with onchocerciasis were recruited by palpation and onchocercomas examined by USG using an ultrasound system equipped with a 7.5 – 10 MHz linear transducer. Onchocercomas were recorded on videotape and evaluated with regard to location, number and size, as well as to movements of adult filariae.

**Results:**

In the 61 patients 303 onchocercomas were found by palpation and 401 onchocercomas were detected by USG. In 18 out of 61 patients (29.5%), altogether 22 nodules with moving adult *O. volvulus *filariae were detected and are presented in animated ultrasound images as mp-4 videos.

**Conclusion:**

Ultrasonographical examinations of onchocercomas where living adult filariae can be displayed may serve as a new tool for the longitudinal observation *in vivo *of patients with onchocerciasis undergoing treatment and as an adjunct to histological evaluation.

## Background

Onchocerciasis is endemic in 37 countries and about 18 million people are infected in Africa and Latin America. Health organisations are aiming to eliminate onchocerciasis as a major public health problem. The Onchocerciasis Control Programme (OCP) has successfully targeted transmission by control of the vector (black fly) but this programme ended in 2002. Within the scope of the African Programme for Onchocerciasis Control (APOC), annual mass treatment with the microfilaricidal drug ivermectin is carried out. However, elimination of transmission has proven more difficult than expected [2]. A recent conference concluded that eradication is not feasible with the present tools [4] and that new drugs are needed. Beside the indirect parameter of microfilariae count, surgical intervention (nodulectomies) is needed to verify effects of drugs on the vitality and fertility of adult *Onchocerca volvulus *after treatment.

Thus, there is an urgent need for the development of more effective drugs that either kill or long-term sterilize the adult worms. In addition, it would be worthwhile to have a non-invasive tool to provide repeatable longitudinal observation of alterations in the vitality and motility of worms induced by potentially macrofilaricidal drugs.

Different to regular detection and longitudinal observation of worm movements (Filaria Dance Sign-FDS) in lymphatic vessels in bancroftian filariasis [1,6,7] motile adult filariae of the species *O. volvulus *in onchocercoma tissue have not been displayed to date by ultrasonography. Therefore the usefulness of USG regarding frequency of detection of adult living *O. volvulus *filariae was evaluated in the present study. The non-invasive USG-method provides on one hand the opportunity for longitudinal observation in terms of number and size of onchocercomas and on the other hand repeatable examinations to observe alterations on motile adult *O. volvulus *filariae *in vivo*.

## Materials and Methods

The study was part of a double blind, placebo controlled treatment study with doxycycline carried out in the Lower Denkyira District in the Central Region of Ghana. This study was approved by the Committee on Human Research and Ethics of the School of Medical Science at KNUST, Kumasi, Ghana. The study conformed to the principles of the Helsinki Declaration of 1975 (as revised1983 and 2000).

### Study site and patients

After detailed explanation about the study in the Ghanaian language Twi, patients were pre-selected by palpation of worm nodules. The number and location of palpated onchocercomas was documented on patient forms. 61 participants, who had not receive antifilarial treatment within the past 2 years, were examined by ultrasonography before receiving doxycycline or placebo treatment. According to the study protocol, all patients also received ivermectin 150 μg ivermectin/kg body weight – 4 months after study start.

To determine microfilaria load before treatment, two skin snips of the upper part of each buttock were taken with a corneoscleral (Holth) punch (Koch, Hamburg, Germany). Each skin snip was weighed with an analytical balance (Sartorius electronic balance, Göttingen, Germany). Microfilarial load was calculated per mg/skin.

The skin snips were incubated at room temperature in 100 μl NaCl 0.9% for 6 – 20 hours in separate wells of a 96-well round bottom microtitre plate (Nunc, Roskilde, Denmark). Microfilariae were counted after incubation using 63-fold magnification of a microscope.

### Ultrasound examination

Ultrasound examinations were performed before treatment started using a SonoSite 180 Plus^® ^(SonoSite Inc.; Washington, USA) system, equipped with an L38 mm 5 MHz – 10 MHz linear transducer. For the actual ultrasound examination of the onchocercomas, frequencies of 7.5 and 10 MHZ were used. A digital camcorder SONY DCR-PC120E PAL Handycam^® ^(Sony Corporation, Japan) was directly connected to the ultrasound machine to record real time videos on mini DV-tapes for documentation of the findings. Patients were examined in a supine position in order to avoid artefacts due to movements. The examiner observed each nodule in longitudinal and transverse scans to detect movements of adult filariae. First the transducer was positioned at the largest diameter of a nodule or at the largest echo-free area in case of cystic nodules. Thereafter, imaging was carried out in panorama-mode to provide more information. Every detected onchocercoma was measured in the two dimensional b-mode for length and width in the largest diameter the examiner could adjust. Nodules were measured in total – including the capsule consisting of connective tissue. The detection of all onchocercomas was recorded on videotape and later compared to the number of nodules detected by palpation. The video documentation was also used to evaluate nodules containing moving worms with regard to number, size, motility, echo-free areas in onchocercomas as sign for necrotic proceedings and acoustic shadowing, reflecting moving and static fragments of the worms.

## Results

### Study participants

The study group consisted of 44 male and 17 female participants. Their age ranged from 19–61 years. All patients were tested for microfilarial load which ranged from 0 – 290 mf/mg skin (geom. mean 11.21 mf/mg). Altogether 356 onchocercomas were palpated, whereas 401 (geom. mean 5.140 nodules / per patient) onchocercomas were detected by USG in the participating 61 patients. In male patients a total of 272 (geom. mean 5.417) nodules were palpated while 303 (geom. mean 6.010) were detected by USG, in female patients 84 (geom. mean 4.487) onchocercomas were palpated and 98 (geom. mean 5.210) were detected by USG (table 1).

**Table 1:** Number of nodules palpated vs. number of nodules detected by ultrasound (USG).

	**Male patients N = 44**	**Female patients N = 17**	**Total N = 61**
USG	303	98	401
Palpation	272	84	356

### Correlation of palpation and USG

Onchocercomas could be clearly differentiated from lymph nodes, where a sinus was visible with afferent and efferent lymphatic vessels, and from lipomas, which appear brighter, more echo dense and homogeneous than onchocercomas. Foreign bodies such as pieces of metal, stones or thorns could be differentiated from onchocercomas due to their sharp signals, which result from their well-defined borders and surrounding granuloma tissues.

All palpated nodules could be detected by USG. In 23 patients the number of nodules detected by palpation matched with the number of onchocercomas detected by USG. In 26 patients more onchocercomas were detected by USG than by palpation. These onchocercomas were of a larger size and tightly packed in a surrounding capsule of connective tissue. By USG the examiner was able to differentiate more precisely the exact number of nodules, divided by septulae of connective tissue inside larger onchocercomas. USG of 12 patients showed less onchocercomas than palpated: 2 lipomas, 2 foreign bodies and 8 lymph nodes had been erroneously judged as onchocercomas by palpation. The 2 painful foreign bodies, which were seen by USG, were excised and verified.

### Reproducibility of USG findings

3 patients underwent USG examination on 3 consecutive days at the first time-point (onset of the study). All onchocercomas were detected at the same locations and judged as onchocercomas. 45 of the 61 study patients underwent a second confirmatory ultrasound examination 4 months after study start. All nodules judged as onchocercomas by USG at the beginning of the study could be rediscovered and still proved to be onchocercomas. 30 out of the 45 USG re-examined patients underwent nodulectomies at this time-point. After nodulectomies no more onchocercomas were visible by USG at locations known to be positive for these findings before.

### Measurement of onchocercomas

The size of the nodules measured by USG varied from 0.18 cm^2 ^to 9.44 cm^2 ^(geom. mean 1.823, median 1.78). Evaluation of the total cross-sectional area (cm^2) ^of all nodules measured per patient showed that nodules were larger in older patients (figure 6). There was no significant relation between age and the occurrence of cystic nodules where worm movements could be documented by USG (Mann-Whitney U test: p = 0.1989). Volume measurements of the onchocercomas were not done since this would have been inaccurate with a 2-dimensional device. Therefore 3-dimensional ultrasound scanners are necessary.

### Patients with onchocercomas containing motile adult filariae

**Figure 1:**
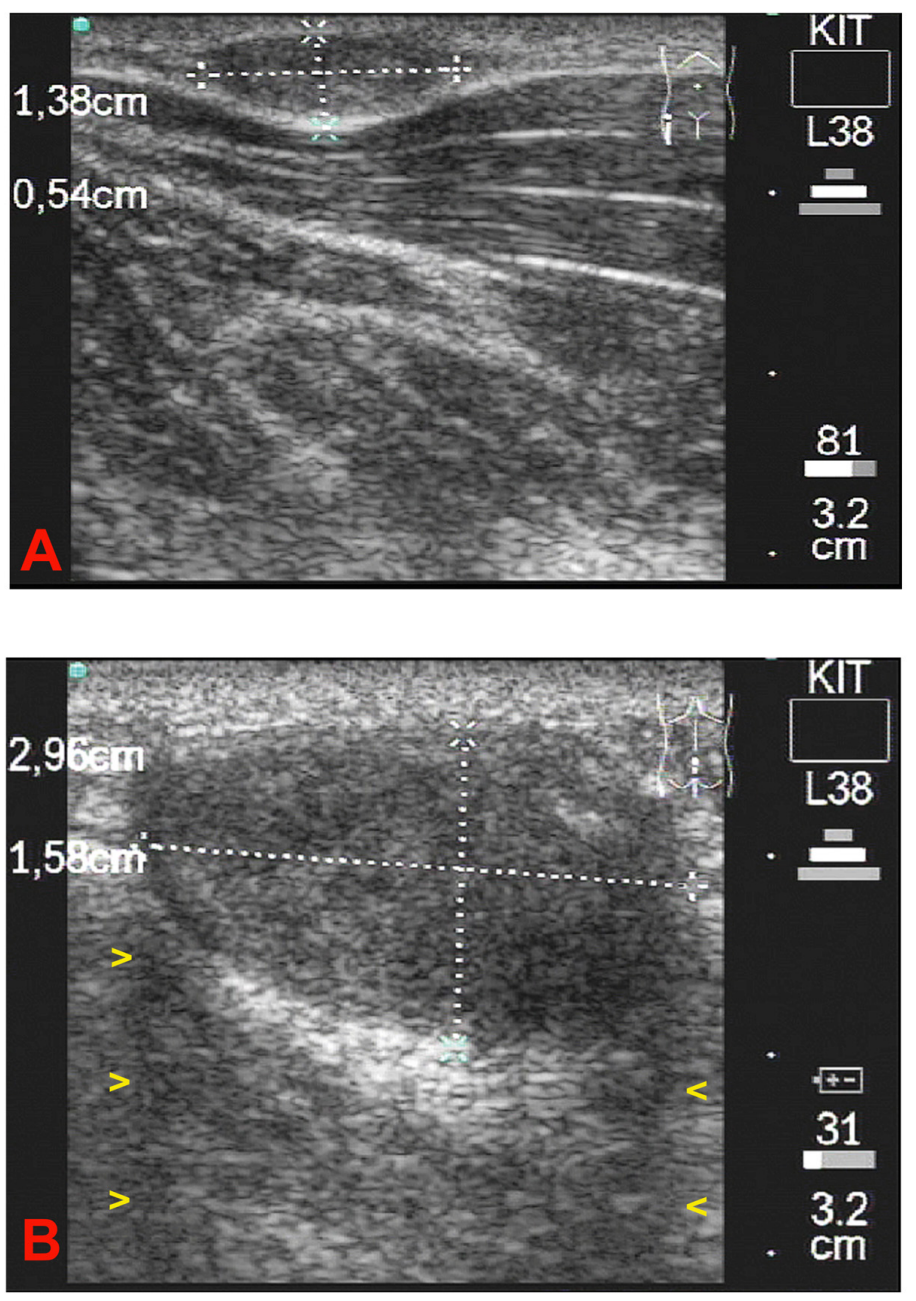
**1A**: Longitudinal scan of the patient's right iliac crest. The b-mode image shows a small subcutaneous onchocercoma. Measurement is shown in the largest transverse and longitudinal section of the nodule. In this homogenous onchocercoma no worm movements were detected. **1B**: Longitudinal scan of the crena analis. A medium sized homogenous subcutaneous onchocercoma can be seen. As in image 1A no worm movements were detected. A differentiation of worm centre, corresponding to a coil of worms, from the capsule was not possible in this nodule. A lateral shadow is visible on both sides of the onchocercoma (yellow arrowheads)

**Figure 2:**
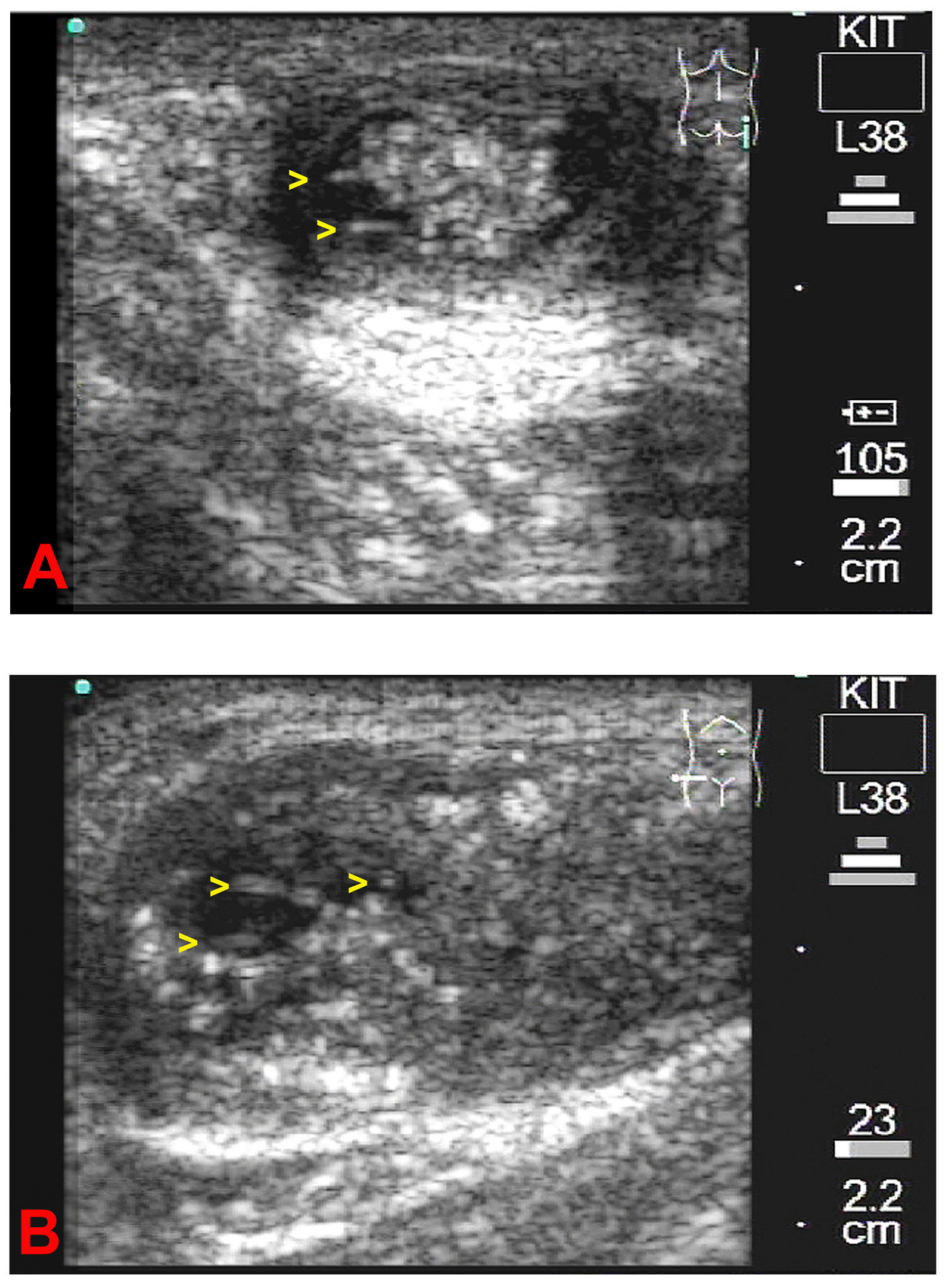
**2A**: Longitudinal scan of the patient's right trochanter. The b-mode image shows a medium sized onchocercoma. The observer can easily differentiate between the worm centre containing a conglomerate of coiled adult filaria (e) and the capsule of the nodule. The worms are surrounded by cystic fluid. The yellow arrowheads are positioned where worm movements were detected. The conglomerate of worms causes a strong back wall reflection behind the onchocercoma. **2B**: Transverse scan of the patient's right iliac crest. A large onchocercoma with a small cystic area (echo-free-black – zone) can be seen. The yellow arrowheads are positioned where worm movements were detected. Fragments of the worm body can be seen as a double layer membrane. The corresponding video image can be seen as Additional File 1.

**Figure 3:**
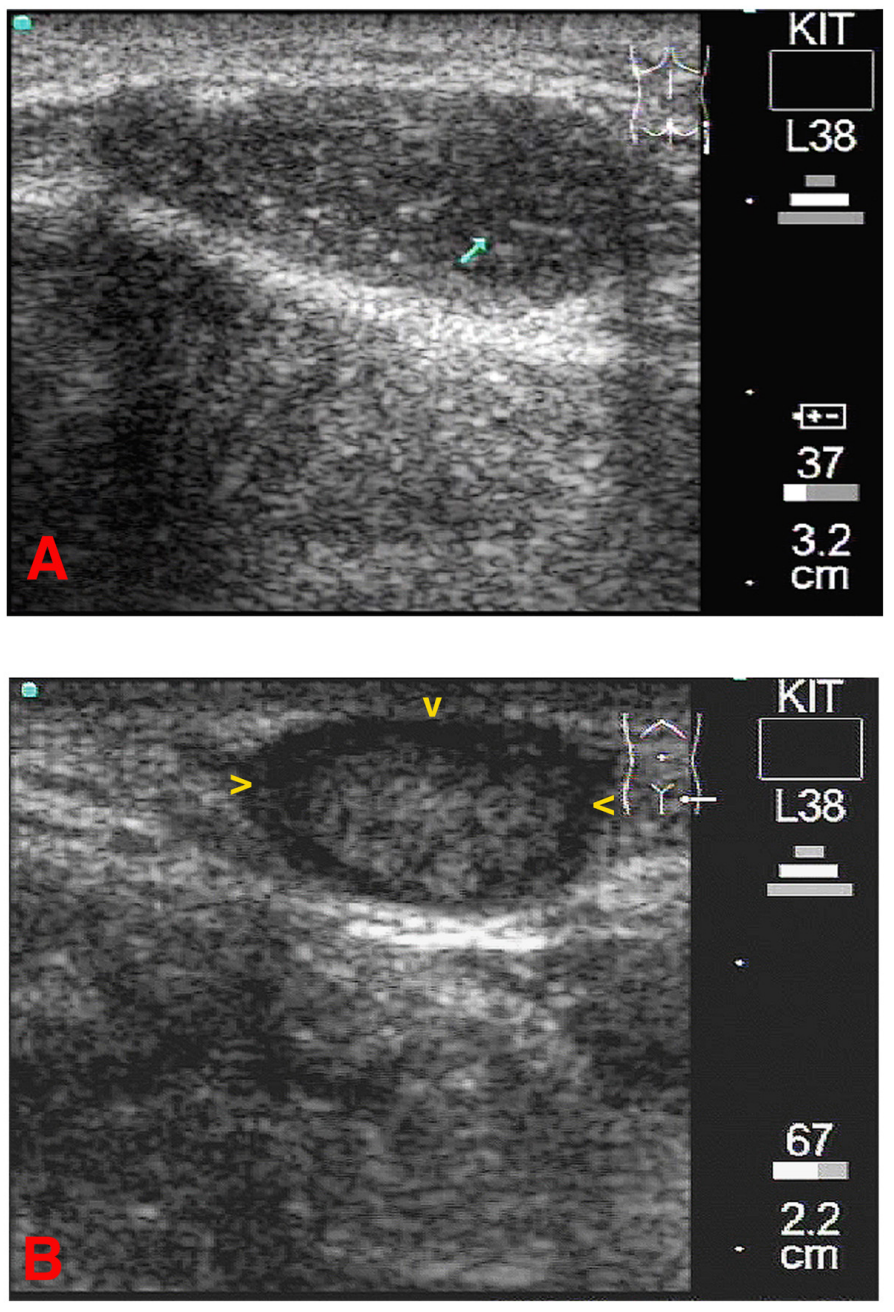
**3A**: Longitudinal scan of the patient's right trochanter. A homogenous medium sized onchocercoma can be seen. Although there is no cystic, echo-free area seen, worm movements were detectable in the b-mode image (blue arrow). Lateral shadows and a back wall reflection are visible. The corresponding video image can be seen as Additional File 2. **3B**: Transverse scan of the patient's left iliac crest. The worm(s) is (are) surrounded by cystic fluid seen as echo-free areas (yellow arrowheads) and can be differentiated from the capsule of the onchocercoma. In this nodule no worm movements could be detected.

**Figure 4:**
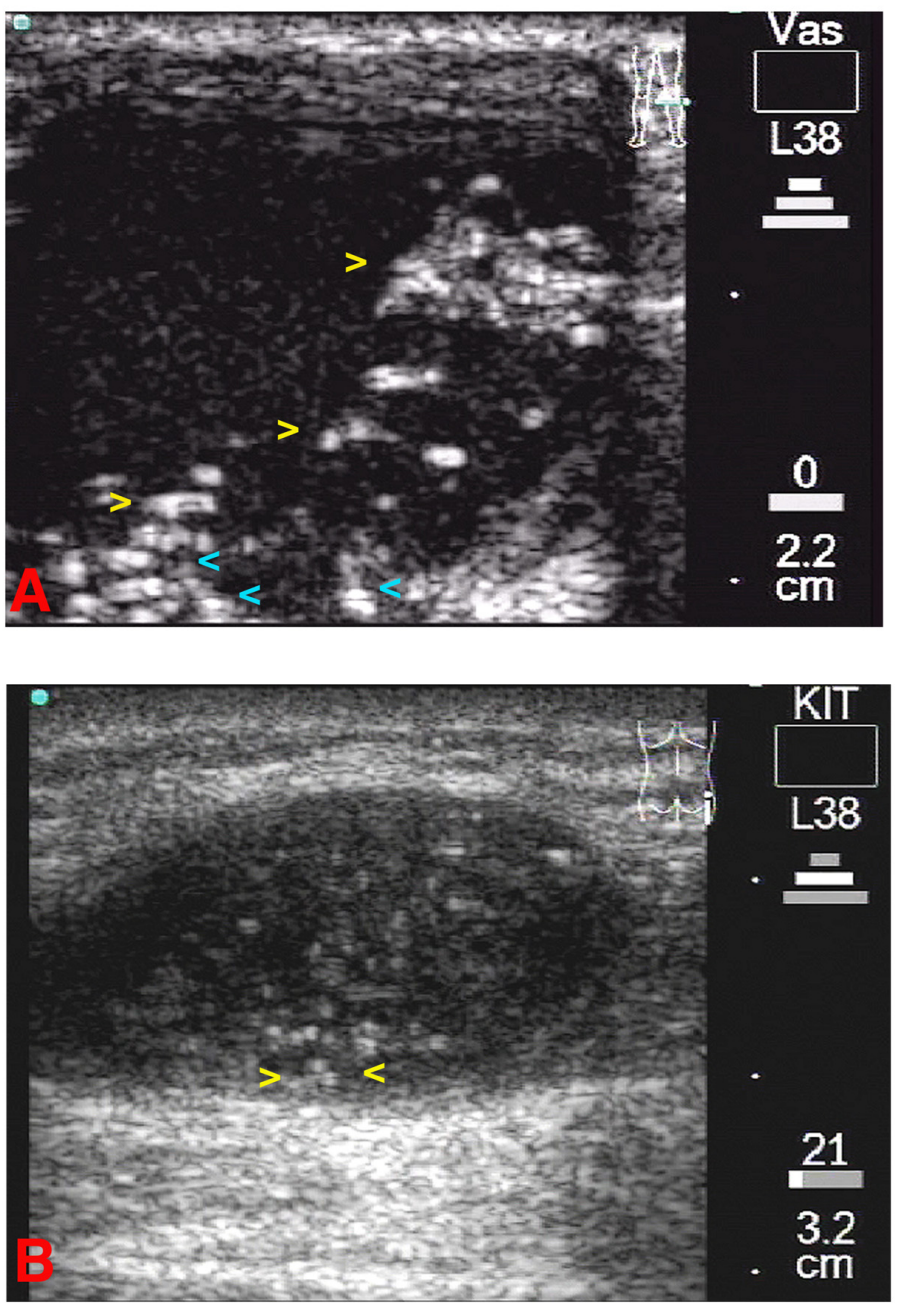
**4A**: Transverse scan of the patient's left knee. A large cystic onchocercoma can be seen. Yellow arrowheads indicate at moving fragments of the worm(s), while the blue arrowheads indicates static fragments of the worm body (ies). The corresponding video image can be seen as Additional File 3. **4B**: Longitudinal scan of the patient's right trochanter. A medium sized cystic nodule where minimal movements were detected. The worm(s) is (are) surrounded by cystic fluid seen as echo-free areas (yellow arrowheads) and can be differentiated from the capsule of the onchocercoma.

In 18 out of 61 (14 male, 4 female) participants (table 2), 22 onchocercomas with moving adult filariae were observed by USG. 15 patients had one nodule, 2 patients had 2 nodules, and 1 patient had 3 nodules with living filariae. Anatomical sites where cystic nodules could be detected were thorax, trochanter, iliac crest, crena analis, knee and foot (heel). The frequency distribution of onchocercomas where worm movements could be observed is shown in table 3. The living adult worms were visible as acoustic enhancement (bright echo) reflected from tissue moving in echo-free areas of onchocercomas (additional file 1,3) and in one case in hypodense nodular tissue (additional file 2). The echo-free parts of the nodule provided a so-called "acoustic window" where worm movements could easily be seen (figure 2A, 2B, 3B, 4A, additional file 1,3) in comparison to nodules where adult worms are packed tightly in a capsule of connective tissue (figure 1A, 1B, 3A, additional file 2). Within the echo-free areas moving adult filariae appeared as coiled and twisted structures moving around each other in the surrounding fluid. The echo-free areas differed from a very small part of the nodule to areas comprising the whole nodule (figure 2A, 2B, 3B, 4A, 4Badditional file 1,3).

**Table 2:** Total numbers of male and female patients with onchocercomas, in which motile or non-motile adult worms were detected by ultrasound (USG).

	**Male patients**	**Female patients**	**Total**
Patients with onchocercomas where adult motile filariae could be detected by USG	**14**	**4**	**18 (29.5%)**
Patients with onchocercomas where no adult motile filariae could be detected by USG	**30**	**13**	**43 (71.5%)**
**Total**	**44**	**17**	**61(100%)**

**Table 3:** Anatomical sites where onchocercomas with living adult filariae occurred:

Anatomical site	Crena analis	Trochanter	Iliac crest	Thorax	Knee	Foot	**Total**
Number	**7**	**5**	**3**	**3**	**3**	**1**	**22 **(observed in 18 patients)

Beside motile adult filariae, all cystic nodules contained parts of probably degenerated fragments of adult worms, seen as particles where the ultrasound beam cannot pass through and is reflected and visible as double layers moving in the cystic fluid (figure 4A, additional file 3).

The Pulse Wave Doppler Technique as described for the detection of adult filariae of *Wuchereria bancrofti *[8,14] was used to visualise movements of living worms of *O. volvulus *as a function of time and frequency, which however appears slow and rare in comparison to adult filariae in lymphatic vessels. Thus the relevance of the Pulse Wave Doppler technique appears less important for the observation and confirmation in onchocerciasis in comparison to examinations in bancroftian filariasis.

### Correlation to histology

A preliminary analysis of onchocercomas by histology, confirmed the results on the proportion of live adult worms from an earlier study (85%)[10], which was based in the same endemic area. The data clearly show that most of non-motile worms are vital but that the adult worms are not detectable and do not appear as motile worms by USG. The reasons may be that i) worms are too tightly packed in host tissue to appear motile; ii) the connective tissue surrounding the onchocercomas is thick and thus reflects the ultrasound beam of the capsule but not of the worms inside.

Out of the 18 patients presenting cystic nodules with motile worms, 8 patients were re-examined after 4 months before the above-mentioned nodulectomies. All cystic nodules could be rediscovered at the same location and still contained moving adult filariae.

**Figure 5:**
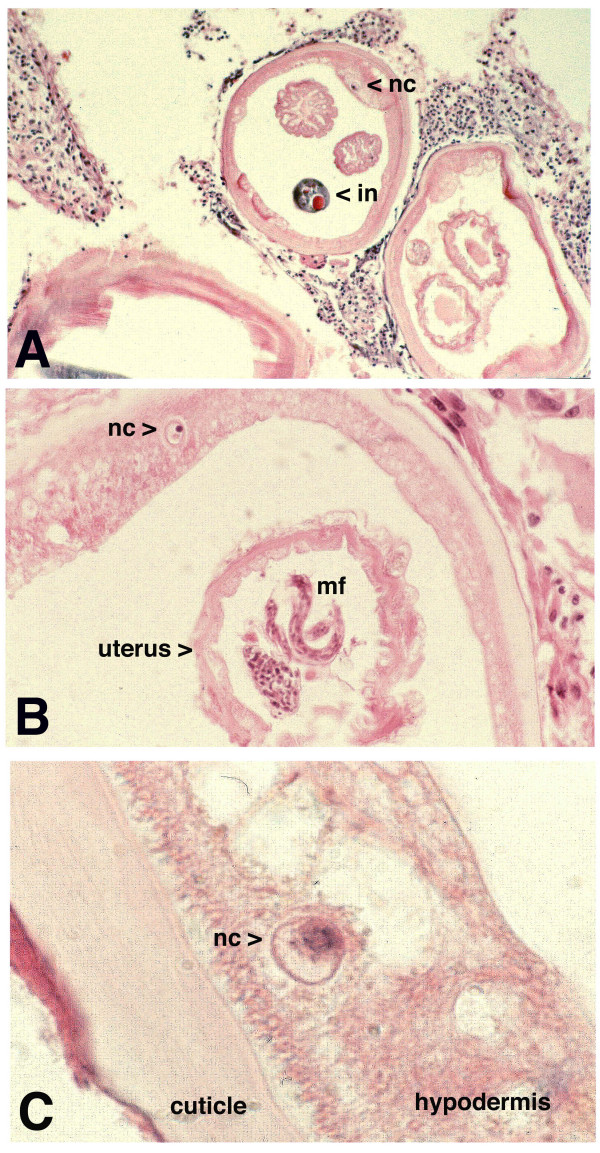
**5A**: Live adult female of *O. volvulus *of a placebo treated patient. A nucleus (nc) in the hypodermis of the body wall (lateral cord) is visible as sign of vitality of the worm. The intestine appears darker beside the two sections of the uterus. (Magnification × 40) **5B**: Section of the same onchocercoma as seen in 5A. The nucleus (nc) is visible in the hypodermis. Intact microfilariae (mf) and pretzel stages (pz) are shown. (Magnification × 60) **5C**: Part of the hypodermis and the cuticle of a female adult *O. volvulus*. The cuticle and the hypodermis appear intact and the nucleus (nc) as sign of vitality is clearly visible. (Magnification × 100).

To correlate these findings in the histology 3 patients presenting nodules with visible movements underwent nodulectomies. In cystic onchocercomas adult filariae were found by histology (figure 5 A–C). A nucleus is visible in the hypodermis of the worms and intact microfilariae are visible inside the uterus as sign of vitality at the time-point of nodulectomy.

**Figure 6:**
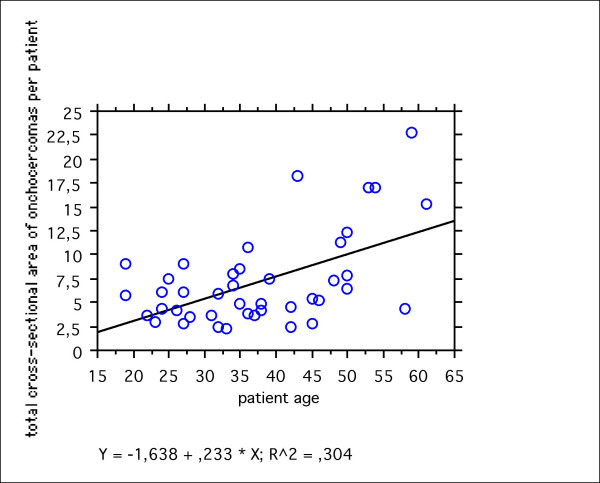
Regression plot of the total cross-sectional area of onchocercomas (detected by USG) in relation to the patient's age. The figure shows that nodules were larger in older patients.

## Discussion

In the present study, onchocerciasis patients were examined by ultrasonography to determine the frequency of detection of living adult *O. volvulus *in onchocercomas. The current study is an improvement over previous attempts to detect worms in onchocercomas as it provides video documentation of worm movements and shows that, with the availability of higher resolution transducers, the frequency of detection of worms containing moving adult worms is surprisingly high. In 18 out of 61 (29.5%) of the examined patients, 22 onchocercomas with moving adult filariae were detected.

Clinical trials to verify potentially macrofilaricidal effects of drugs currently lack methods for repeatable long-term observation of adult living filariae in onchocercomas *in vivo*. The use of ultrasonography to observe onchocercomas has been rare, since movements of adult worms embedded in nodules surrounded by connective tissue were difficult to image. In fact there is only one case listed in the literature where moving adult worms were detected [5]. Homeida et al. [11] first described the use of USG to detect onchocercomas in patients. Poltera and Zak [16] published in 1988 an ultrasound study on onchocercomas, where they described the ability to use USG to observe alterations in nodule tissue after treatment in vitro. Leichsenring et al. [12] evaluated in 1990 the use of ultrasound examinations on patients with regard to a suitable tool for longitudinal observation under treatment conditions, however did not show movements of worms. The higher sensitivity of nodule detection by USG in comparison to palpation and the ability to differentiate between onchocercomas and lipomas, lymph nodes and foreign bodies has been reported [12]. In 1994 Darge et al. [5] described that they observed distinct worm movements in 1 of 249 detected nodules of their patient group in Liberia.

An animated documentation of moving adult filariae of *O. volvulus *by video has not been published so far. Due to the improved sensitivity of current ultrasound machines as well as digital video technique we were able to detect and record in almost 30% of the participants of the study 1 to 3 onchocercomas containing moving, adult filariae of *O. volvulus*. Although it has been observed (Prof. Buettner, Bernhard Nocht Institute of Tropical Medicine Hamburg, personal communication) that onchocercomas seem to become cystic over the course of time, and that worms die from their end of the body while the prosoma and the head of the worm keep moving in cystic fluid, we did not find a correlation between the occurrence of moving worms in nodules and age of the patients (18–61 years, Mann-Whitney U test: p = 0.1989). This is probably due to the fact that in our study area, transmission is still high despite the onset of ivermectin mass treatment and therefore, even younger patients already displayed a considerable number of cystic nodules. It might be possible that the number of cystic nodules is higher in areas where there have been several rounds of MDA resulting in reduced transmission and thus, over-aged nodules.

Effects of drugs are currently verified by histological examinations of the nodules after minimal invasive surgery (nodulectomy). Worms can be evaluated with regard to number of male and female worms, size, embryogenesis and spermiogenesis [3]. Immunohistology is used to determine whether worms were alive before nodulectomy. To this, sections of nodules can be stained e.g. with antisera against *O. volvulus *GST1 (Glutathione-S-transferase) and *Yersinia *hsp 60 (heat shock protein), the latter cross reacting with mitochondrial hsp 60 in the worms [10,13]. GST1 and mitochondrial hsp 60 are only produced by living worms and appear as a strong staining in the hypodermis.

The evaluation of treatment by the use of (immuno-) histology has the disadvantage that there is only one single time point for examination, which cannot be repeated and cannot be carried out at several different time points. In contrast, the use of USG offers a non-invasive, cost effective tool, which permits observation at many time points but has the disadvantage that adult worms of *O. volvulus *are coiled around each other and it seems not possible to determine their number and their sex, although the male worms are only 3–5 cm long in comparison to female filariae with a length of 30–80 cm.

USG is not a surrogate for immunohistology. With USG it is impossible to evaluate embryogenesis and spermatogenesis of adult filariae. Furthermore, USG can provide qualitative, not quantitative information about worm number in nodules. Another disadvantage of USG is that only a fraction of worms that prove "vital" in histology is able to display movements either due to dense host tissue, or due to echo dense connective tissue surrounding the onchocercoma. Nevertheless, although the number of onchocercomas where motile worms could be detected appears to be few (5.5%), the fact that 29.5% of patients are carriers of cystic nodules with motile filariae offers the possibility for long-term observation of the vitality of worms in vivo in a reasonable number of infected humans. Therefore, information provided by USG may prove useful for the evaluation of potentially macrofilaricidal drugs and has the advantage of being non-invasive. The latter point is important, as the compliance of the patients is much better in cases where a painless method is used.

## Conclusion

Ultrasonographical examination of cystic onchocercomas containing living adult filariae may serve as a new tool in the longitudinal observation of patients with onchocerciasis as an adjunct to histological evaluation. USG may also facilitate clinical trials in terms of observation of macrofilaricidal activities with regard to effects that may have been overlooked before (e.g. repeated high dose ivermectin treatment).

## Competing interests

The author(s) declare that they have no competing interests.

## Authors' contributions

S. Mand recruited patients, did the ultrasound examinations, compiled the data and wrote the paper draft. Y. Marfo-Debrekyei, A. Debrah and L Batsa carried out the skin snips and the microfilaridermia counting. They performed patient management during recruitment and ultrasound examinations. M. Buettner recruited patients, performed physical examinations, and carried out the treatment. K. Pfarr helped in manuscript preparation and offered constructive comments. O. Adjei did preparatory studies for proper selection of villages, negotiations with the village elders, and performed the ethical clearance. A. Hoerauf designed and supervised the study, participated with patient recruitment and ultrasound examinations, compiled the data and edited the final manuscript version.

## Supplementary Material

Additional File 1 Transverse scan of the patient's right iliac crest. A large onchocercoma with a small cystic area (echo-free – black – zone) can be seen. Within this cystic area movements of the adult filaria(e) are visible. Fragments of the worm body can be seen as a double layer membrane. The corresponding image can be seen as figure 2B. Click here for file

Additional File 2 Longitudinal scan of the patient's right trochanter. A homogenous medium sized onchocercoma can be seen. Although there is no cystic – echo-free area, worm movements are presented in the b-mode image (arrow). Lateral shadows and a back wall reflection are visible. The corresponding image can be seen as figure 3A. Click here for file

Additional File 3 Transverse scan of the patient's left knee. A large cystic onchocercoma can be seen. Movements of a conglomerate of coiled adult filariae are displayed in the cystic fluid of the nodule. Static fragments of the worms are visible in the lower left part of the video image. The corresponding image can be seen as figure 4A. Click here for file
